# Probiotic *Lactobacillus* Species Strengthen Intestinal Barrier Function and Tight Junction Integrity in Experimental Necrotizing Enterocolitis

**DOI:** 10.4172/2329-8901.1000159

**Published:** 2017-01-02

**Authors:** Brian P Blackwood, Carrie Y Yuan, Douglas R Wood, Joseph D Nicolas, Justyna S Grothaus, Catherine J Hunter

**Affiliations:** 1 Ann and Robert H. Lurie Children’s Hospital of Chicago, Department of Pediatric Surgery, Chicago, Illinois, USA; 2 Northwestern University Feinberg School of Medicine, Department of Pediatrics, Chicago, Illinois, USA

**Keywords:** *Lactobacillus*, Necrotizing enterocolitis, Tight junctions, Barrier function, Probiotic

## Abstract

Necrotizing enterocolitis (NEC) is a serious intestinal disease that occurs in newborn infants. It is associated with major morbidity and affects 5% of all infants admitted to neonatal intensive care units. Probiotics have variable efficacy in preventing necrotizing enterocolitis. Tight junctions (TJ) are protein complexes that maintain epithelial barrier integrity. We hypothesized that the probiotics *Lactobacillus rhamnosus* and *Lactobacillus plantarum* strengthen intestinal barrier function, promote TJ integrity, and protect against experimental NEC. Both an *in vitro* and an *in vivo* experimental model of NEC were studied. Cultured human intestinal Caco-2 cells were pretreated with *L. rhamnosus* and *L. plantarum* probiotics. TJ were then disrupted by EGTA calcium switch or LPS to mimic NEC *in vitro*. Trans-epithelial resistance (TER) and flux of fluorescein isothiocynate dextran was measured. TJ structure was evaluated by ZO-1 immunofluorescence. *In vivo* effects of ingested probiotics on intestinal injury and ZO-1 expression were assessed in a rat model of NEC infected with *Cronobacter sakazakii* (CS). Caco-2 cells treated with individual probiotics demonstrated higher TER and lower permeability compared to untreated cells (p<0.0001). ZO-1 immunofluorescence confirmed TJ stability in treated cells. Rat pups fed probiotics alone had more intestinal injury compared with controls (p=0.0106). Probiotics were protective against injury when given in combination with CS, with no difference in intestinal injury compared to controls (p=0.21). Increased permeability was observed in the probiotic and CS groups (p=0.03, p=0.05), but not in the probiotic plus CS group (p=0.79). *Lactobacillus* sp. strengthened intestinal barrier function and preserved TJ integrity in an *in vitro* experimental model of NEC. *In vivo*, probiotic bacteria were not beneficial when given alone, but were protective in the presence of CS in a rat model of NEC.

## Introduction

Necrotizing enterocolitis (NEC) is an inflammatory intestinal disorder that affects premature infants. Despite years of research, it remains the most common gastrointestinal emergency seen in the neonatal intensive care unit (NICU) [1]. NEC is associated with an average mortality of 15–30%, but may be as high as 75–85% in its most severe forms [2,3]. Survivors may require surgical resection of necrotic bowel and incur associated morbidities, including short gut syndrome, growth delay, and neurodevelopment disorders [4]. The pathophysiology of NEC is not well understood and as a result, care is mostly supportive with no defined preventative therapy available. There currently are no recommended strategies that consistently prevent NEC [5,6]. The possibility of administering probiotic species to protect at-risk infants from developing NEC is a captivating concept that has received significant attention [7].

Probiotics are defined as “live microorganisms that provide beneficial health effects on the host when administered in adequate amounts” [8]. Probiotics have been postulated to improve gastrointestinal health by promoting intestinal motility, increasing the production of trefoil proteins and mucin, and enhancing degradation of food protein antigens. Probiotic species may compete against pathogenic microorganisms for nutrition or epithelial binding; however, none of these hypotheses have been definitively proven [9,10]. In fact, several researchers have noted concern that probiotics may themselves be harmful [11]. *Lactobacillus* sp. have been isolated from many different types of infective lesions as well as blood stream infections [12]. Additionally, probiotic bacteria have been found in the blood of patients with NEC who received prophylactic dosing [13]. This suggests that the probiotics themselves may not be as benign as originally thought and may have adverse effects on the patient receiving the prophylactic dose.

Although probiotics may modulate gut pathophysiology via multiple mechanisms, compelling data suggests that probiotics alter the expression of epithelial tight junctions (TJ) [14,15]. TJ are a type of cell-to-cell adhesion found in the apical portion of intestinal epithelial cells that provide a primary barrier for the intracellular space [16]. These adhesion structures are made up of organized protein complexes at the cell membrane [17]. TJ proteins may become internalized and/or degraded during injury and stress, and some evidence from mouse models of NEC suggests that probiotics can help stabilize TJ and protect them from injury [18]. Past research has highlighted how specific probiotics appear to be protective against TJ disruption [19,20]. Additional studies have demonstrated that specific probiotics affect TJ integrity; however, these studies have not evaluated TJ integrity in the context of NEC [21,22].

Several clinical trials have investigated the use of prophylactic probiotic species in the treatment of NEC, however there is insufficient data to warrant a change in practice or to support guidelines for the use of the probiotics prophylactically [23,24]. *Lactobacillus rhamnosus* and *Lactobacillus plantarum* are two probiotic species which have been analyzed in human NEC studies [25]. Additionally, both *L. rhamnosus* and *L. plantarum* have been identified as immunobiotic and confer protection against intestinal injury [26]. We hypothesized that *L. rhamnosus* and *L. plantarum* will alter intestinal barrier function and TJ integrity, but will also protect against experimental NEC.

## Materials and Methods

### Bacterial strains

The *Cronobacter sakazakii* [24] clinical strain BAA-894 (American Type Culture Collection (ATCC), Manassas, VA, USA), was grown at 37°C in Luria broth [27], centrifuged at 3000 rpm to pellet down the bacteria, and washed twice in saline before being added to cultures or formula to induce NEC in rats. *L. rhamnosus* (ATCC 53103) [28] [25] and *L. plantarum* (ATCC 10241) were cultured in MRS media overnight culture to a density of 10^8^ CFU/mL before being used as described in the experiments. The final concentration of bacteria in experiments was 10^7^ CFU/mL.

### Cells

The human intestinal epithelial cell line, Caco-2 (ATCC, Manassas, VA), was grown in DMEM/F12 and 5% fetal bovine serum (FBS). Cells were cultured on 24-well, 6.5-mm Transwells (0.4 μm polycarbonate) (Corning, Sigma-Aldrich, St. Louis, MO, USA) until transepithelial membrane resistance reached 250 ohms/cm^2^.

### Reagents for membrane disruption

Lipopolysaccharide (LPS) from *Escherichia coli* clinical strain 0111:B4 (Sigma-Aldrich) was stored at 4°C. LPS was dissolved in sterile 0.9% normal saline [VWR, Radnor, PA, USA] to achieve a stock concentration of 10 mg/ml. EGTA (Bioworld, Dublin, OH, USA) was diluted in media and a dose response curve was performed using 1 mM, 3 mM, and 5 mM concentrations.

### Caco-2 transepithelial resistance [TER] measurements

TER was measured with a voltohmmeter (EVOM2; World Precision Instruments, Sarasota, FL, USA). Once Caco-2 cells had established TJ, as indicated by a TER value of at least 850 ohms/cm^2^, cells were pretreated with *Lactobacillus* sp., added to the apical surface of the cells at a concentration of 10^7^ CFU/ml. Controls were not exposed to probiotics. TJ were then disrupted by either adding 1 mg/ml of LPS to the basal layer of each well [29,30] or by a calcium switch protocol in which 1 mM, 3 mM, or 5 mM EGTA was added to each well [27,31,32]. At 1 hour and 2 hours after treatment, TER was measured. Control [untreated] cells were used at each time point and all measurements of TER were normalized to this value.

### Caco-2 FITC dextran permeability measurements

Caco-2 cells were pretreated with *Lactobacillus* sp. and then challenged with either LPS or EGTA. One or 2 hours after addition of LPS or EGTA, 3 kDa fluorescein isothiocynate (FITC)-labelled dextran was added to the apical layer [29]. After 2 hours, the basal layer was then collected and assayed in triplicate. A fluorescent plate reader (Molecular Devices GeminiXS; Sunnyvale, CA, USA) was used to assay the concentration of FITC dextran in the basal layer, which was compared to the concentration initially applied to the apical layer. A control [untreated group] was used at all-time points and experiments were normalized to these groups. All cell culture experiments were performed in biological triplicate and repeated three times.

### Caco-2 zona occludens 1 (ZO-1) immunofluorescence

After exposure of Caco-2 cells (control or pretreated with *Lactobacillus* sp. for 2 hours) to LPS or EGTA for 5 hours, the cells were processed for immunofluorescence analysis to visualize the subcellular location of TJ proteins. Caco-2 grown on transwell membranes were washed and then fixed with 1% paraformaldehyde. Cells were blocked with phosphate buffered saline (PBS)/Triton-X and 10% normal goat serum. The membranes were incubated with a primary antibody against ZO-1 (Invitrogen, Carlsbad, CA) at 4°C overnight. The membrane was washed four times in PBST and then blocked in secondary antibody conjugated with Alexa Fluor 488 (Invitrogen) at room temperature for 1 hour. The membranes were then mounted with Fluoroshield with DAPI (F6057; Sigma-Aldrich) and examined under a fluorescent microscope. The mean fluorescence intensity was measured with ImageJ and differences between groups were compared by ANOVA.

### Animals

Approval for all animal experiments was obtained from the International Animal Care and Use Committee of Northwestern University. Timed-pregnant Sprague Dawley rats were purchased from Charles River Laboratories (Kalamazoo, MI, USA) and induced near-term at E21 with a subcutaneous injection of Pitocin 0.1 Units. Newborn rat pups were collected and separated into experimental groups. The pups were subject to gavage formula feeding (15 g Similac 60/40 (Abbott, Abbott Park, IL) in 75 ml of Esbilac canine milk replacer (Pet-Ag Inc., Hampshire, IL)) for a total volume of 0.25–0.35 ml three times daily for 4 days. Pups were exposed to hypoxia (5% O_2_, 95% N_2_) for 5 minutes twice daily in a modular chamber (Billups-Rothenberg Inc, Del Mar, CA, USA). Experimental groups included clean formula controls (Clean) (n=42); a probiotic formula group containing the *Lactobacillus* sp. [Pro] (n=42); a group with just CS bacteria in the formula (n=38) [24]; and a group with *Lactobacillus* sp. and CS bacteria in the formula (Pro+CS) (n=42). On postnatal day 4, the rat pups were gavage fed 40 mg of FITC-labelled dextran per 100 g of body weight. Two hours after FITC feeding, the pups were euthanized. Pups were euthanized before postnatal day 4 or if they displayed clinical symptoms of NEC (abdominal distention and discoloration) or respiratory distress. Animals were housed in the Northwestern University facilities that are fully accredited by the Association for Assessment and Accreditation of Laboratory Animal Care International. Animals were provided with environmental enrichment. All procedures and protocols were approved by Northwestern University Institutional Animal Care and Use Committee and were conducted in accordance with guidelines set forth by the Guide for the Care and Use of Laboratory Animals.

### Histological analysis

Tissue samples and blood samples were collected from the animals after euthanasia for analysis. NEC was graded microscopically by a pediatric pathologist blinded to groups, from grade 0 (normal) to 3 (severe) on the basis of pathological manifestations including submucosal edema, epithelial sloughing, hemorrhage, neutrophil infiltration, derangement of intestinal villus architecture, intestinal perforation, and necrosis. Grade 0 corresponds with normal architecture and healthy appearing villi. Grade 1 has some mild evidence of inflammation without derrangement of villus achitecture or inflammatory cell infiltrate. Grade 2 is consistent with experimental necrotizing enterocolitis and has evidence of disruption of normal villi, sloghing and inflammatory cell infiltrate. Grade 3 is characterized by loss of villi and histiological evidence of perforation.

### Rat pup FITC dextran permeability measurements

As described above, postnatal day 4 rat pups from each group were fed 40 mg of 10 kDa FITC dextran per 100 g body weight. After 2 hours, the pups were euthanized and a blood sample was collected. The serum/enteral ratio of FITC dextran was measured. This number was then averaged within each experimental group for comparison. The blood sample was analyzed with a fluorescent plate reader to measure the concentration of FITC dextran, which was then compared to the concentration given in the feed to assess intestinal permeability.

### Rat pup zona occludens-1 immunofluorescence

On postnatal day 4, rat pups were euthanized and intestinal segments were collected. The intestines were preserved in optimal cutting temperature media (O.C.T) and then cut into 4 μm sections. The tissue was washed with Phosphate-buffered saline [PBS] pH 8.0 and fixed with 1% paraformaldehyde. Tissue sections were blocked with PBS 0.1% Triton-X and 10% normal goat serum and then incubated in PBS with the ZO-1 primary antibody 1:500 [Invitrogen]. The sections were washed 4 times in PBST followed by incubation with secondary antibody, Alexa Fluor 488 Goat Anti-Rabbit IgG Antibody (Invitrogen). Sections were mounted with Fluoroshield with DAPI (Sigma-Aldrich) and examined with a Nikon A1R confocal microscope. The mean fluorescence intensity was measured from 3 different pup samples with 6 different slides per condition. ImageJ was used for imaging and differences between groups were compared by ANOVA.

### Rat pup intestinal segment protein extraction

Rat pups intestine tissue samples were isolated and suspended in Allprotect Tissue Reagent (Qiagen Inc., Valencia, CA, USA) or flash frozen in liquid nitrogen before being stored at −80°C. The frozen tissue was sectioned and suspended in lysis buffer (Cell Signaling Tech, Boston, MA, USA) containing 1 mM Phenylmethylsulfonyl fluoride (PMSF). Samples were homogenized for 3 minutes on ice. After centrifugation for 1 minute at 4°C, the supernatents were removed and stored at −80°C. To isolate proteins from cellular monolayers grown on 100 mm plates (5.5 × 10^6^ cells), the media was removed and 1 mL of PBS was added and the cells were scraped and transferred to a microfuge tube. Samples were microfuged at 5,000 rpm at 4°C for 10 minutes and the supernatents were removed. The cell pellets were resuspended in 500 μl of lysis buffer (as above). The mixture was then drawn three times through a 27-gauge needle and gently mixed on a rotating platform for 30 minutes at 4°C followed by centrifugation at 4°C for 15 minutes at 10,000 rpm. The supernatants were removed and stored at −80°C. Before use, the tissue and cellular samples were thawed on ice. A total of 5X Laemmli SDS sample buffer was added and then boiled for 3 minutes. The samples were stored at −20°C until used.

### Immunoblot analysis of ZO-1

Protein expression of ZO-1 in the rat pup intestinal segments was measured by immunoblot. Intestinal protein samples were vortexed and 10 μl were electrophoresed in 8% SDS-PAGE and then transferred onto nitrocellulose membranes (Bio-Rad, Hercules, CA). The membrane was then blocked in 5% Blotting Grade Blocker (Bio-Rad, Hercules, CA) and PBS with 0.05% Tween for 2 hours. The membranes were incubated overnight with rabbit anti–ZO-1 (Invitrogen) at a concentration of 1:500 at 4°C, washed three times with PBS/Tween before addition of the secondary antibody (goat anti-rabbit (Santa Cruz, Santa Cruz, CA)). The membrane was developed in Western Blotting Detection Reagent (Amersham, Arlington Heights, IL) for 5 minutes before being transferred to film. Band densities were measured using Image Lab Software (Bio-Rad, Hercules, CA).

### Statistical analysis

Graphs were generated using Excel and GraphPad Prism 6 software (La Jolla, CA). Statistical analysis (ANOVA or Student’s *t* test) was performed using GraphPad Prism 6. Differences were considered significant at p<0.05.

## Results

### *Lactobacillus* sp. increase barrier resistance in an *in vitro* Caco-2 cell model of NEC

**Figure 1:**
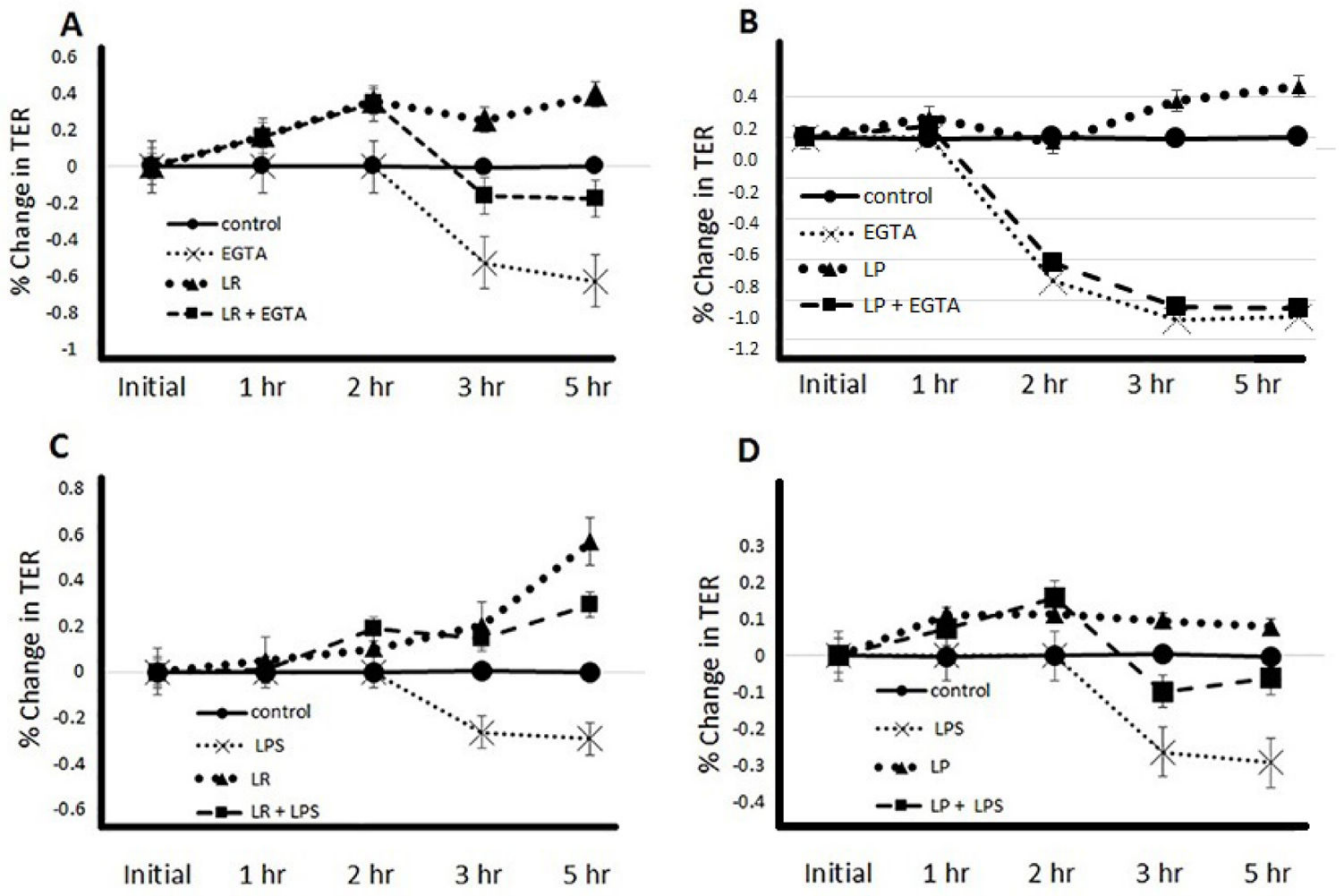
LR and LP pretreatment protects against EGTA- and LPS-induced decreases in barrier resistance. Caco-2 cells were untreated (Control) or pretreated for 2 hours with probiotics (LR–panels A and C or LP–panels B and D) before undergoing TJ disruption by EGTA calcium switch (panels A and B) or LPS treatment (panels C and D). TER was measured at each time point after treatment with membrane disrupting reagents. (A) LR pretreatment increased TER in the absence (p<0.0014) and presence of EGTA (p<0.0001). (B) LP pretreatment also increased TER in the absence (p<0.0059) and presence of EGTA (p<0.0009) as compared to those cells treated with EGTA alone. (C) LR pretreatment increased TER in the absence (p<0.0001) and presence of LPS (p<0.042). (D) LP pretreatment had less of an effect on TER in the absence (p<0.043) and presence of LPS (p<0.047).

To determine the effect of *Lactobacillus* sp. on barrier function, we pretreated Caco-2 enterocytes with either LR or LP for 2 hours prior to LPS or EGTA treatment. Controls were not treated with probiotics, EGTA, or LPS. Both LPS and EGTA cause intestinal barrier disruption, and provide a useful *in vitro* model of NEC [29]. TER was used as a measure of membrane barrier resistance and TJ disruption. TER was monitored throughout pretreatment with the probiotics, and then again after the addition of the membrane-disrupting reagents [15]. A significant and continuous reduction in TER throughout the 5-hour time course was seen following treatment of enterocytes with either EGTA or LPS Figure 1.

In the experiments with EGTA, TER in the enterocytes significantly increased after treatment with LR (p<0.0014; Figure 1A) or LP (p<0.0059; Figure 1B), and the detrimental effect of EGTA on TER was reversed with pretreatment with LR (p<0.0001 compared to EGTA alone; Figure 1A). In a similar fashion, enterocytes treated with LP demonstrated similar changes with a significant difference noted between the EGTA-treated cells and the LP + EGTA Cells (p<0.0009) Figure 1B. In the LPS model, again, there was a significant increase in TER when enterocytes were pretreated with LR (p<0.0001; Figure 1C) and to a lesser extent LP (p=0.043; Figure 1D). Pretreatment with LR was protective against the detrimental effect of LPS on TER (p<0.042, Figure 1C); a similar protective effect of LP was less robust (p=0.047; Figure 1D).

Taken together, probiotic pretreatment increased Caco-2 enterocyte TER compared to the control group and protected against the barrier disruption by EGTA or LPS. LR appeared to provide a greater degree of protection against EGTA- or LPS-mediated injury than did LP.

### *Lactobacillus* sp. decrease membrane permeability in an *in vitro* Caco-2 cell model of NEC

**Figure 2:**
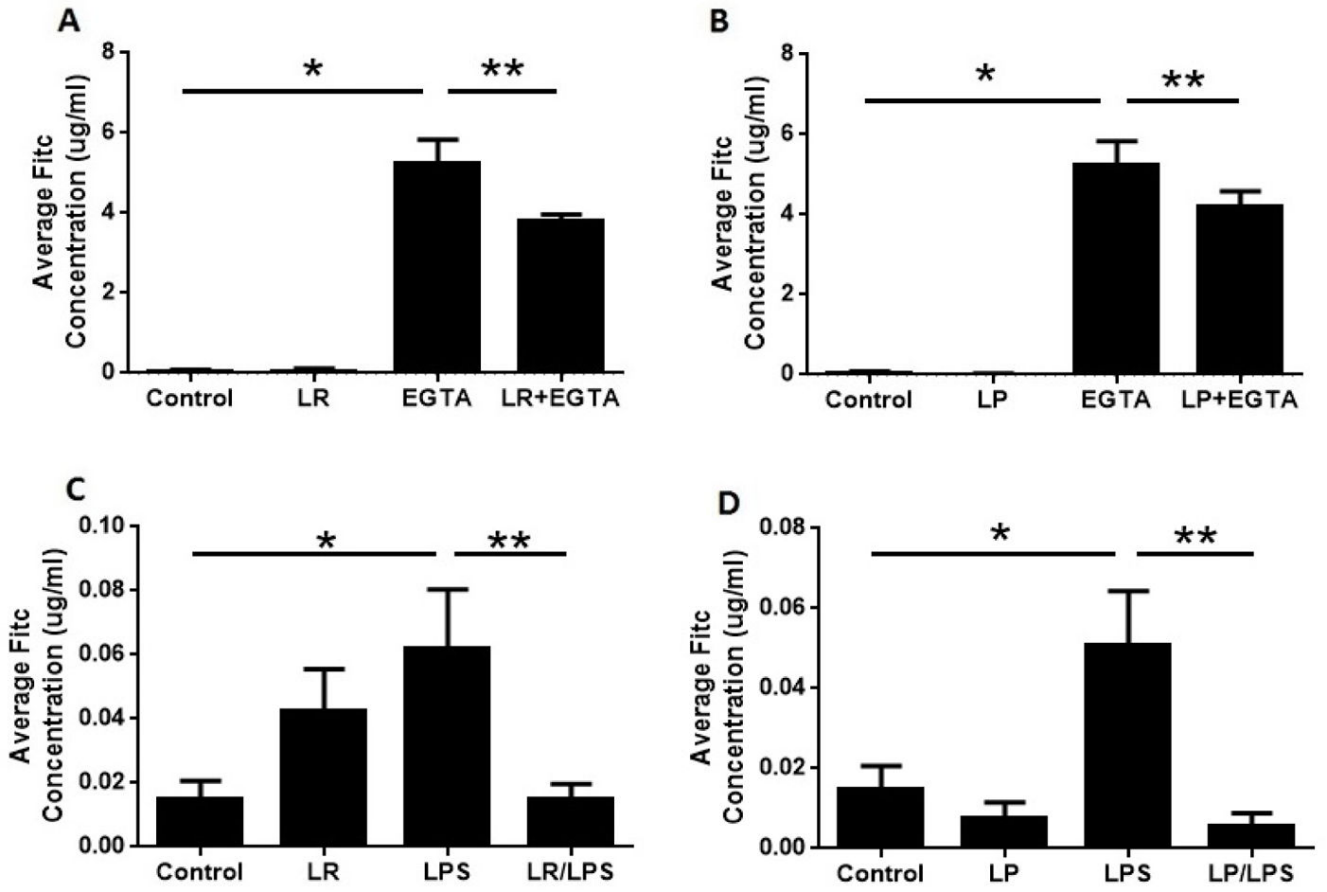
LR and LP pretreatment protects against EGTA- and LPS-mediated increases in FITC permeability. Caco-2 cells were untreated (Control) or pretreated for 2 hours with probiotics (LR–panels A and C; or LP–panels B and D) before undergoing TJ disruption by EGTA calcium switch (panels A and B) or LPS treatment (panels C and D). FITC dextran was added to the apical layer and FITC dextran in the basal layer was measured after 2 hours. (A) EGTA increased permeability compared to Control (^*^p<0.0001); the effect of EGTA was blunted by pretreatment with LR (^**^p<0.0001). (B) EGTA increased permeability compared to Control and LP-treated cells (^*^p<0.0123); the effect of EGTA was decreased by LP pretreatment (^**^p<0.0001). (C,D) LPS significantly increased permeability compared to Control or probiotic-pretreated cells (for LR, ^*^p<0.024 and for LP, ^*^p<0.024). The effect of LPS was blunted upon pretreatment of cells with either LR (^**^p<0.015) or LP (^**^p<0.005).

To determine the effect of *Lactobacillus* sp. on membrane permeability, we applied FITC dextran to the apical layer of Caco-2 cells after a 2 hour pretreatment with LR or LP probiotics, followed by LPS or EGTA treatment. Control cells were untreated. We then measured the concentration of FITC dextran in the basal layer and compared it to that applied to the apical layer to assess membrane permeability. There was a significant decrease in membrane permeability after pretreatment of the cells with probiotics, suggesting a “strengthening” of the barrier. In the presence of EGTA, a significant increase in membrane permeability was seen compared to untreated controls and LR-treated cells (p<0.0001); this effect of EGTA was blunted in cells pretreated with LR (p<0.0001, LR+EGTA compared to EGTA alone; Figure 2A). A similar protective effect of LP pretreatment was seen in cells treated with EGTA (Figure 2B). LPS treatment also increased permeability compared to LR-pretreated or Control cells [p<0.0024], and LR pretreatment blunted the effect of LPS (p<0.015; Figure 2C) [28]. Similarly, LPS increased permeability compared to LP-pretreated or Control cells [p<0.0024] and LP pretreatment decreased the effect of LPS (p<0.005; Figure 2D).

### *Lactobacillus* sp. stabilize TJ in an *in vitro* Caco-2 cell model of NEC

**Figure 3:**
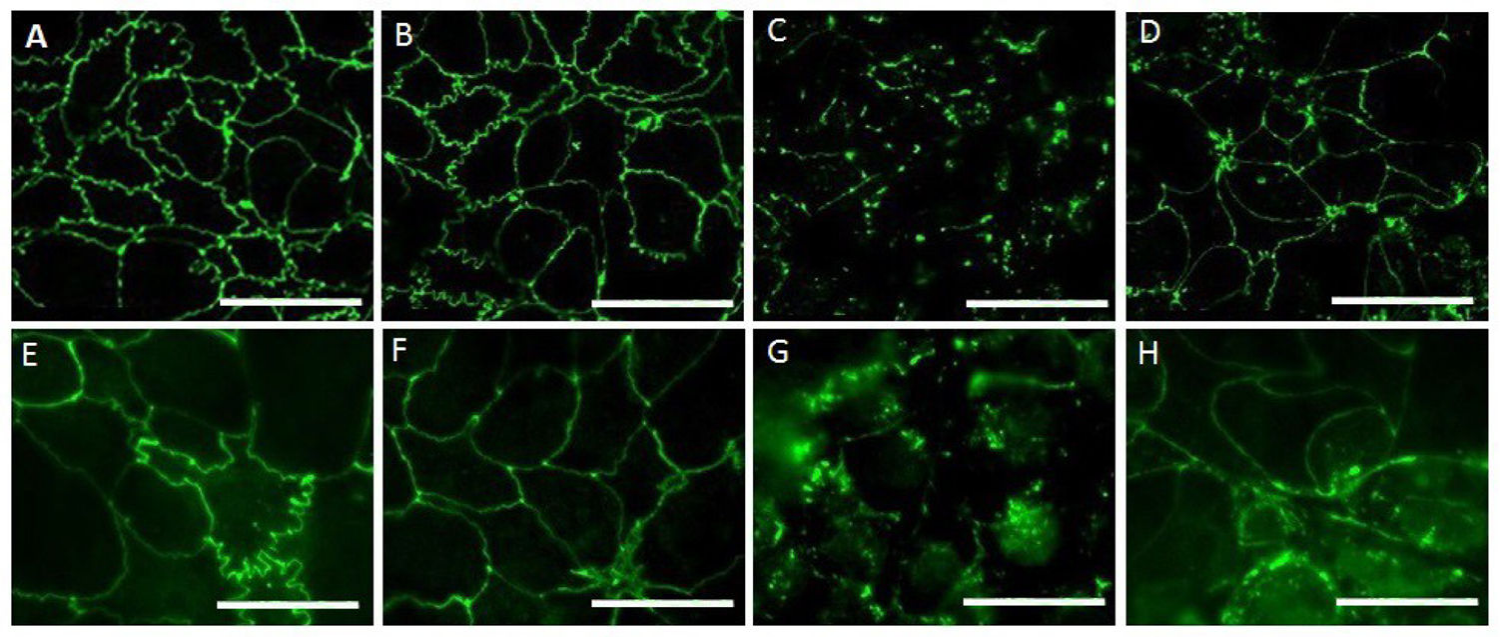
LR and LP are protective against TJ disruption by EGTA. Caco-2 cells were untreated (Control) or pretreated for 2 hours with probiotics (LR or LP) before undergoing TJ disruption with EGTA calcium switch. TJ integrity was evaluated based on immunofluorescent staining of the TJ protein ZO-1 (32) at the end of the 5-hour treatment period. Representative images from 3 samples performed in triplicate are shown for each of the groups of Caco-2 cells: (A) Control, (B) LR-pretreated, (C) EGTA-treated, (D) LR-pretreated + EGTA-treated, (E) Control, (F) LP-pretreated, (G) EGTA-treated cells, (H) LP-pretreated + EGTA-treated (I). The experiment was repeated with LPS rather than EGTA. While TJ disruption based on ZO-1 immunofluorescence was not visually evident in the LPS-treated cells, mean fluorescence intensity (MFI) for ZO-1 was lower in the LPS-treated cells compared to the control cells (p=0.008). Bar=40x.

**Figure 4:**
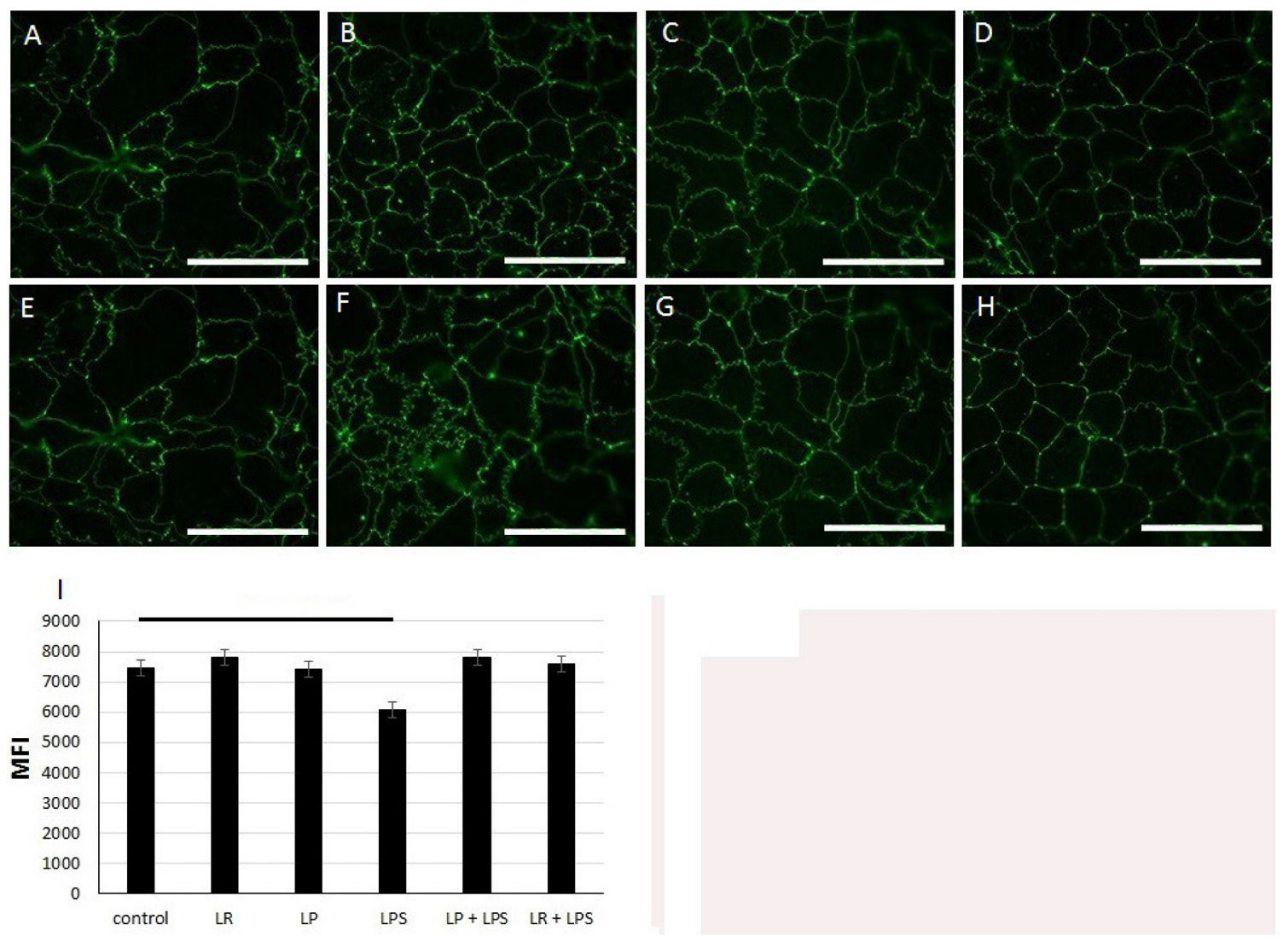
Rat pups with experimental NEC that received LR probiotic had lower intestinal injury scores and decreased FITC permeability compared to controls. Experimental NEC was induced in rat pups. On postnatal day 4, the pups were gavage-fed FITC dextran 2 hours before intestinal segments were collected for histology and injury scoring. (A–D) Representative histological images of intestinal segments from rats fed (A) Clean formula, (B) probiotic alone (Pro), (C) CS alone (CS), (D) and probiotic + CS (Pro + CS). (E) When compared to the rats in the Clean group, the probiotic alone (Pro; ^*^p=0.0106) and CS groups (^**^p=0.002) showed greater intestinal injury, but the Pro + CS group did not (p=0.21). Additionally, the Pro + CS group had a significantly decreased injury score as compared to the CS group (^***^ p=0.009). (F) Greater permeability to FITC dextran was seen in both the Pro (^*^p=0.03) and CS groups (^**^p=0.05) but not in the Pro + CS group (p=0.79) compared to the Clean group. N=40 rat pups. Bar=40x.

Immunofluorescent staining of ZO-1 was used to assess the stability of TJ in response to probiotic pretreatment and EGTA and LPS treatment. Untreated control Caco-2 cells demonstrated normal organized TJ staining patterns (Figure 3A). As expected, EGTA resulted in TJ disruption as seen by diffuse ZO-1 immunofluorescent staining at the end of the 5-hour of treatment period Figure 3C. Interestingly, when either LR or LP was applied as a pretreatment before the cells were exposed to EGTA, there was far less TJ disruption Figure 3D–H. TJ disruption was also evident in Caco-2 cells treated with LPS, though the differences in ZO-1 immunofluorescence staining among the treatment groups were more subtle than those seen with EGTA Figure 4. The calculated MFI [mean fluorescent intensity] demonstrated a significant decrease in intensity in those cells exposed to LPS compared to control cells (p=0.008; Figure 4A). There was no difference in MFI between control cells or any of the LR or LP pretreatment groups, suggesting that probiotic pretreatment prevented LPS-induced barrier disruption Figure 4B–H and ^★^.

### *Lactobacillus* sp. reduce intestinal epithelial damage in an *in vivo* rat pup model of NEC

**Figure 5:**
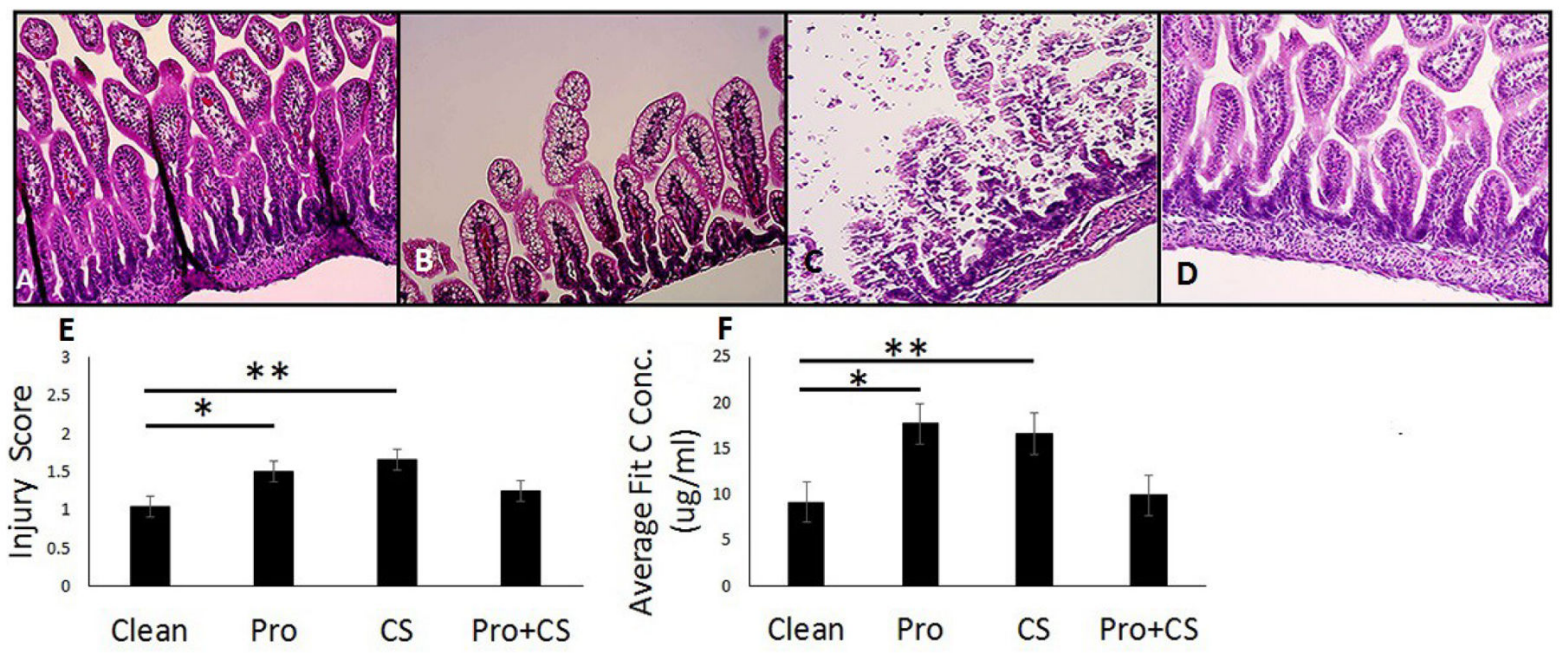
Rat pups that received both LR and CS treatments have higher ZO-1 expression on immunofluorescence. Experimental NEC was induced in rat pups. On postnatal day 4, intestinal segments were collected. (A–D) Representative images of ZO-1 immunofluorescence (32) in the intestine of rats receiving Clean (A), Probiotic (B), CS (C), or Pro + CS (D) formulas. (E) MFI analysis revealed an increase in ZO-1 levels in the Pro + CS group (^*^p=0.05) compared to all other groups. N=40 rat pups. Bar=40x.

To assess whether probiotics prevent experimental NEC *in vivo*, we used our described rat pup model of NEC [33]. NEC rats received CS alone or CS with *Lactobacillus* sp. probiotics in the formula for four days. Other groups of rats received clean formula (Clean) or *Lactobacillus* sp. probiotics for four days; all rats were exposed to hypoxic conditions. After the treatment period, rat pups were sacrificed and tissue was collected for analysis. When compared to the control pups, we found significantly greater intestinal injury in both the probiotic alone (p=0.0106; Figure 5A,B and 5E) and the CS alone groups (p=0.0002; Figure 5C and 5E). However, the pups that received probiotics and CS in combination appeared to have less intestinal injury Figure 5D than the probiotic alone and CS alone groups, and there was no significant difference when compared to controls (p=0.21; Figure 5E).

### *Lactobacillus* sp. decrease membrane permeability in an *in vivo* rat pup model of NEC

To assess the effects of the *Lactobacillus* sp. on intestinal membrane permeability, FITC dextran was gavage fed to the pups two hours prior to sacrifice. Consistent with the histology findings, we found that there was a significant increase in permeability in both the probiotic group (p=0.03; Figure 5F) and the CS group (p=0.05; Figure 5F) compared with control pups. In contrast, the Pro+CS group did not demonstrate an increase in permeability (p=0.79; Figure 5E. These results suggest that an increase in biodiversity may contribute to improve intestinal health.

### *Lactobacillus* sp. stabilize ZO-1 and the membrane barrier in an *in vivo* rat pup model of NEC

**Figure 6:**
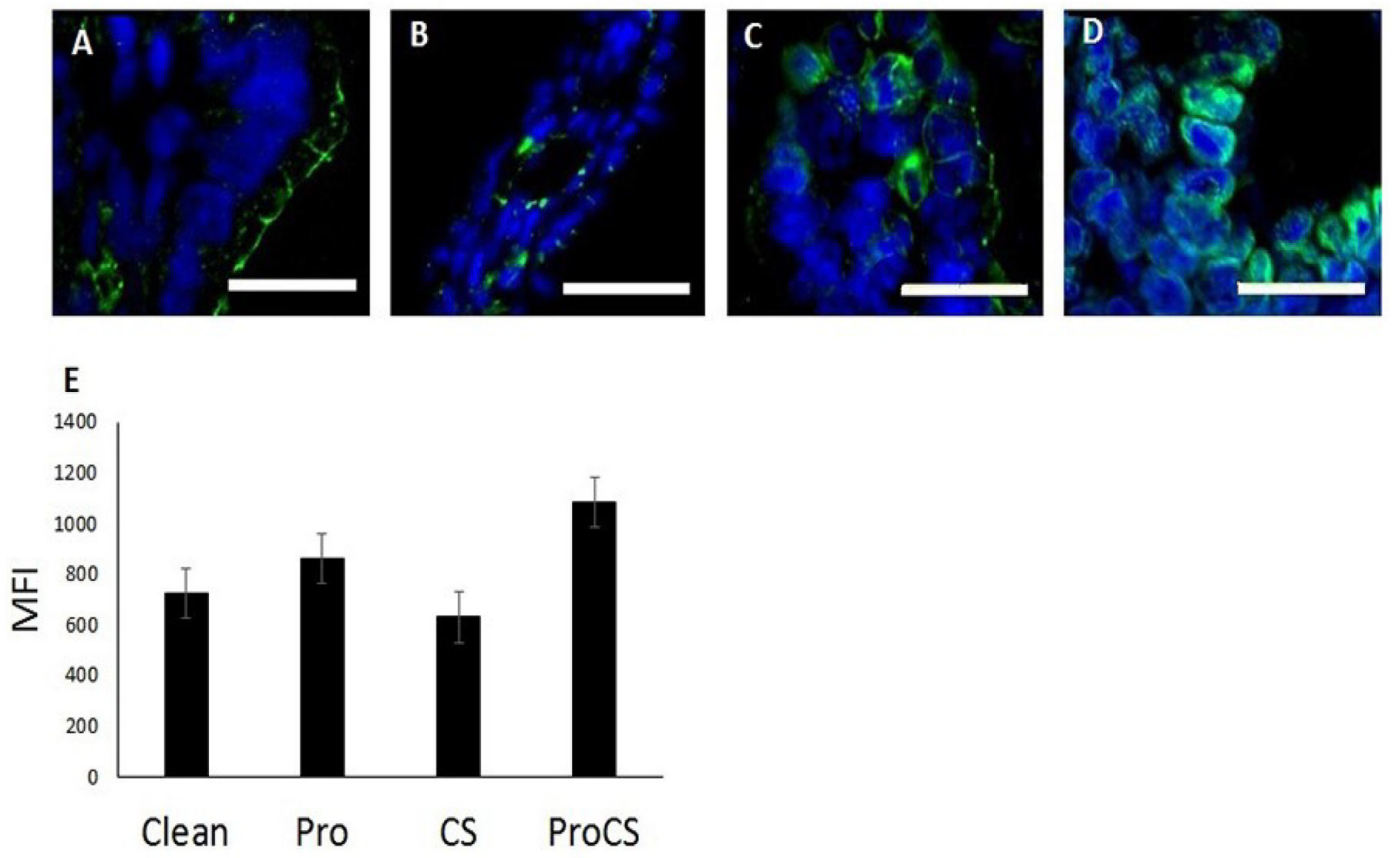
Immunoblot analysis reveals higher ZO-1 expression in rat pups that received LR + CS as compared to those that received CS alone. Experimental NEC was induced in rat pups. On postnatal day 4, intestinal segments were collected. Immunoblot analysis revealed an increase in ZO-1 expression in the Pro + CS rat pups compared to those that received CS alone (p=0.002). N=8–10 rat pups per group.

In order to analyze the effects of *Lactobacillus* sp. on the TJ and membrane barrier in the *in vivo* model, we performed immunofluorescence staining of ZO-1 in the intestine of treated rat pups. Rat pups from the Pro+CS group had significantly higher ZO-1 expression compared to pups in the clean formula group (p=0.05; Figure 6A–E).

**Figure 7:**
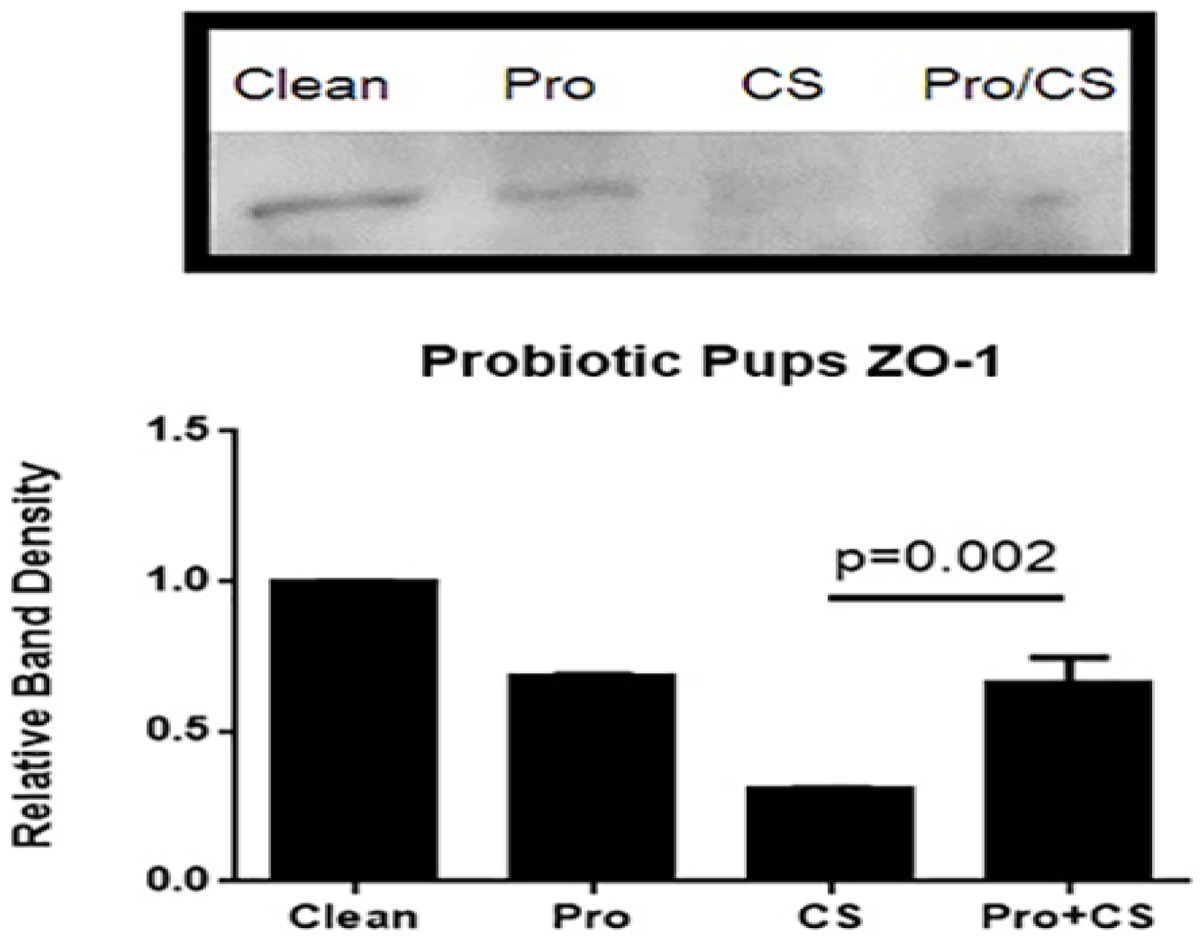
There is no different than expression levels in pups receiving LR alone.

Immunoblot analysis of ZO-1 confirmed the differences in TJ protein expression in response to *Lactobacillus* sp. pretreatment with or without CS. There was significantly greater ZO-1 expression in pups that received LR in addition to CS as compared to rat pups that received CS alone, but was no different than expression levels in pups receiving LR alone Figure 7. Taken together, these results support the idea that probiotics may be most beneficial in a setting of increased microbial diversity and that the mechanism of action by which the probiotics elicit their effect may be in the regulation of the TJ, specifically the ZO-1 protein.

## Discussion

As improvements in medical technology and scientific advances continue to occur, more neonates born prematurely are surviving birth, leading to an increase in the number of infants at risk for developing NEC [34,35]. As a result, NEC has become the most common gastrointestinal emergency in the newborn [1]. Even though NEC has been studied extensively over the past few decades, the mechanisms by which this disease affects its victims remains unknown and as a result there are few treatment strategies available beyond supportive care [3,36]. Although a few clinical studies have shown promising results, currently there is insufficient evidence to recommend a change in practice with regard to prophylactic use of probiotics to prevent NEC [24]. A Cochrane review revealed the benefits of probiotics in neonates, but no clear recommendations can be made given the current data [23]. Until there is a better understanding of how probiotics elicit their effects in the setting of NEC, and of any potentially harmful effects of the probiotics themselves, treatment for NEC will remain mostly supportive and a significant mortality will persist. Furthermore, there are concerns regarding the safety of probiotic administration to the immature intestine [11,13]. The gap in our understanding of NEC and the effects of probiotics on the intestinal barrier is worth further study.

TJ provides an important primary barrier for the intracellular space [16]. TJ are made up of protein complexes found in the cell membrane [17]. There is growing evidence that TJ proteins become internalized as intestinal epithelial cells incur injury, such as that seen in NEC [18]. ZO-1 is a protein that is found in intestinal epithelial TJ and it is important in the scaffolding and structure of the TJ [37]. There is evidence of decreased expression of these TJ proteins in intestinal epithelial cells in NEC and other inflammatory bowel diseases [38,39].

Our data indicates that it is within these intestinal epithelial cell-to-cell adhesion complexes, the TJ, where the *Lactobacillus* probiotics are acting and altering the progression of NEC. The probiotics appear to strengthen and secure the structure of TJ, which in turn improves barrier function. This effect was supported by both our *in vitro* and *in vivo* results.

When the *Lactobacillus* sp. were applied to the cells, there was a significant increase in the TER compared to untreated control, and probiotic pretreatment was also able to blunt the decrease in TER caused by EGTA or LPS membrane disruption. While LR did have a greater effect than LP, both significantly increased the TER. The difference in effect seen between the two probiotic species may indicate a slightly different mechanism of action or potency. This is an interesting finding that warrants future investigation.

Using TER as a marker for TJ integrity, both LR and LP strengthened cell-to-cell adhesion as compared to the control cells and appeared to prevent membrane disruption. This strengthening of cell adhesion and prevention of membrane disruption was further characterized by immunofluorescence staining of ZO-1. Similar findings were seen when we evaluated the Caco-2 cell barrier function in our FITC dextran experiment. Enterocytes that received a pretreatment of LR or LP demonstrated increased membrane integrity and improved barrier function.

Similar findings were seen *in vivo* in our rat pup NEC model, with a slight variation. In the rat pup model, we used injury scores, immunofluorescence of ZO-1, and immunoblot analysis of ZO-1 as markers for the membrane integrity, as well as FITC dextran to evaluate permeability. When we looked at the injury scores, we found that the LR did protect the rat pups from NEC, but it was only protective when CS was present. Both the probiotic and CS groups had higher injury scores compared to the control group, but intestinal injury was not seen in the group that received both probiotics and CS. We believe that the probiotics are still acting on the tight junctions in a similar way as in the *in vitro* model, but in the *in vivo* model, an increase in biodiversity appears to be important. Our data may suggest that probiotics alone do not necessarily promote intestinal epithelial health and that they may in fact cause intestinal injury. The background microbial environment into which the probiotics are being placed may be of significant importance in determining the efficacy of the probiotics. There have been reports of the benefits of a more diverse microbiome in the gut and this interaction appears to affect the efficacy of the probiotics in other *in vivo* models [40–42].

Immunofluorescence and immunoblot analysis confirmed that TJ expression was higher when *Lactobacillus* was present along with the CS bacteria as compared to those pups that received CS alone. FITC dextran measurements in the pups to assess membrane permeability and barrier function showed similar findings to the injury scores. There was higher permeability to FITC dextran in both the LR and the CS groups, indicating injury; however, there was no increase in permeability in the probiotic plus CS group when compared to the control group. This further leads us to believe that the LR is beneficial in the presence of the CS bacteria, which is often found in outbreaks of infant NEC [43,44].

In conclusion, we have shown that *Lactobacillus* probiotic species strengthen intestinal barrier function and tight junction integrity in both an *in vitro* and an *in vivo* model of NEC. Of the probiotic species we have studied, LR appears to be the most protective; however, our results highlight the fact that a unicellular *in vitro* model may produce different results from the more complex *in vivo* model. In the *in vitro* Caco-2 model, we saw a clear benefit from the probiotic treatments (LR and LP), but the *in vivo* model produced more complex results. In the *in vivo* model, the protective qualities of the probiotic appeared to only occur when the CS bacteria was present, suggesting that a more diverse microbiome is beneficial for intestinal health. Our *in vivo* data also give credence to the idea that the probiotics themselves may cause harm to the intestinal epithelial cells and as a result, clinicians should be cautious in their delivery of prophylactic probiotics.

## References

[1] Kosloske AM (1994). Epidemiology of necrotizing enterocolitis. Acta Paediatr Suppl.

[2] Hunter CJ, Podd B, Ford HR, Camerini V (2008). Evidence vs experience in neonatal practices in necrotizing enterocolitis. Journal of Perinatology: Official Journal of the California Perinatal Association.

[3] Lin PW, Nasr TR, Stoll BJ (2008). Necrotizing enterocolitis: recent scientific advances in pathophysiology and prevention. Seminars in perinatology.

[4] Blakely ML, Lally KP, McDonald S, Brown RL, Barnhart DC (2005). Postoperative outcomes of extremely low birth-weight infants with necrotizing enterocolitis or isolated intestinal perforation: a prospective cohort study by the NICHD Neonatal Research Network. Annals of surgery.

[5] Group E (2010). Incidence of and risk factors for neonatal morbidity after active perinatal care: extremely preterm infants study in Sweden (EXPRESS). Acta paediatrica.

[6] Schanler RJ, Lau C, Hurst NM, Smith EO (2005). Randomized trial of donor human milk versus preterm formula as substitutes for mothers’ own milk in the feeding of extremely premature infants. Pediatrics.

[7] Deshpande G, Rao S, Patole S, Bulsara M (2010). Updated meta-analysis of probiotics for preventing necrotizing enterocolitis in preterm neonates. Pediatrics.

[8] FAO/WHO Health and nutritional properties of probiotics in food including powder milk with live lactic acid bacteria. Report of the Joint Food and Agriculture Organization (FAO) of the United Nations/World Health Organization (WHO) Expert.

[9] Jacobsen CN, Rosenfeldt Nielsen V, Hayford AE, Moller PL, Michaelsen KF (1999). Screening of probiotic activities of forty-seven strains of Lactobacillus spp. by in vitro techniques and evaluation of the colonization ability of five selected strains in humans. Applied and Environmental Microbiology.

[10] Manzoni P, Mostert M, Leonessa ML, Priolo C, Farina D (2006). Oral supplementation with Lactobacillus casei subspecies rhamnosus prevents enteric colonization by Candida species in preterm neonates: a randomized study. Clinical Infectious Diseases: an official publication of the Infectious Diseases Society of America.

[11] Doron S, Snydman DR (2015). Risk and safety of probiotics. Clinical infectious diseases: an official publication of the Infectious Diseases Society of America.

[12] Ishibashi N, Yamazaki S (2001). Probiotics and safety. The American journal of clinical nutrition.

[13] Zbinden A, Zbinden R, Berger C, Arlettaz R (2015). Case series of Bifidobacterium longum bacteremia in three preterm infants on probiotic therapy. Neonatology.

[14] Thuijls G, Derikx JP, de Haan JJ, Grootjans J, de Bruine A (2010). Urine-based detection of intestinal tight junction loss. Journal of Clinical Gastroenterology.

[15] Anderson RC, Cookson AL, McNabb WC, Park Z, McCann MJ (2010). Lactobacillus plantarum MB452 enhances the function of the intestinal barrier by increasing the expression levels of genes involved in tight junction formation. BMC Microbiology.

[16] Tsukita S, Furuse M, Itoh M (2001). Multifunctional strands in tight junctions. Nature Reviews Molecular Cell Biology.

[17] Mitic LL, Van Itallie CM, Anderson JM (2000). Molecular physiology and pathophysiology of tight junctions I. Tight junction structure and function: lessons from mutant animals and proteins. American Journal of Physiology Gastrointestinal and Liver Physiology.

[18] Bergmann KR, Liu SX, Tian R, Kushnir A, Turner JR (2013). Bifidobacteria stabilize claudins at tight junctions and prevent intestinal barrier dysfunction in mouse necrotizing enterocolitis. The American Journal of Pathology.

[19] Ewaschuk JB, Diaz H, Meddings L, Diederichs B, Dmytrash A (2008). Secreted bioactive factors from Bifidobacterium infantis enhance epithelial cell barrier function. American Journal of Physiology Gastrointestinal and Liver Physiology.

[20] Ohland CL, Macnaughton WK (2010). Probiotic bacteria and intestinal epithelial barrier function. American Journal of Physiology Gastrointestinal and Liver Physiology.

[21] Miyauchi E, O’Callaghan J, Butto LF, Hurley G, Melgar S (2012). Mechanism of protection of transepithelial barrier function by Lactobacillus salivarius: strain dependence and attenuation by bacteriocin production. American journal of physiology Gastrointestinal and liver physiology.

[22] Sultana R, McBain AJ, O’Neill CA (2013). Strain-dependent augmentation of tight-junction barrier function in human primary epidermal keratinocytes by Lactobacillus and Bifidobacterium lysates. Applied and environmental microbiology.

[23] AlFaleh K, Anabrees J (2014). Probiotics for prevention of necrotizing enterocolitis in preterm infants. Evidence-based child health: a Cochrane review journal.

[24] Mihatsch WA, Braegger CP, Decsi T, Kolacek S, Lanzinger H (2012). Critical systematic review of the level of evidence for routine use of probiotics for reduction of mortality and prevention of necrotizing enterocolitis and sepsis in preterm infants. Clinical nutrition.

[25] Fernandez-Carrocera LA, Solis-Herrera A, Cabanillas-Ayon M, Gallardo-Sarmiento RB, Garcia-Perez CS (2013). Double-blind, randomised clinical assay to evaluate the efficacy of probiotics in preterm newborns weighing less than 1500 g in the prevention of necrotising enterocolitis. Arch Dis Child Fetal Neonatal Ed.

[26] Tada A, Zelaya H, Clua P, Salva S, Alvarez S (2016). Immunobiotic Lactobacillus strains reduce small intestinal injury induced by intraepithelial lymphocytes after Toll-like receptor 3 activation. Inflamm Res.

[27] Klingler C, Kniesel U, Bamforth SD, Wolburg H, Engelhardt B (2000). Disruption of epithelial tight junctions is prevented by cyclic nucleotide-dependent protein kinase inhibitors. Histochemistry and cell biology.

[28] Ulreich S, Foster KW, Stier SA, Rosenfield AT (1980). Acute cholecystitis. Comparison of ultrasound and intravenous cholangiography. Arch Surg.

[29] Hirotani Y, Ikeda K, Kato R, Myotoku M, Umeda T (2008). Protective effects of lactoferrin against intestinal mucosal damage induced by lipopolysaccharide in human intestinal Caco-2 cells. Yakugaku zasshi: Journal of the Pharmaceutical Society of Japan.

[30] Sheth P, Delos Santos N, Seth A, LaRusso NF, Rao RK (2007). Lipopolysaccharide disrupts tight junctions in cholangiocyte monolayers by a c-Src-, TLR4-, and LBP-dependent mechanism. American journal of physiology Gastrointestinal and liver physiology.

[31] Citi S (1992). Protein kinase inhibitors prevent junction dissociation induced by low extracellular calcium in MDCK epithelial cells. The Journal of cell biology.

[32] Anderson DJ, Podgorny K, Berrios-Torres SI, Bratzler DW, Dellinger EP (2014). Strategies to prevent surgical site infections in acute care hospitals: 2014 update. Infection control and hospital epidemiology.

[33] Hunter CJ, Singamsetty VK, Chokshi NK, Boyle P, Camerini V (2008). Enterobacter sakazakii enhances epithelial cell injury by inducing apoptosis in a rat model of necrotizing enterocolitis. The Journal of infectious diseases.

[34] Gane B, Bhat BV, Adhisivam B, Joy R, Prasadkumar P (2014). Risk factors and outcome in neonatal necrotising enterocolitis. Indian journal of paediatrics.

[35] Guthrie SO, Gordon PV, Thomas V, Thorp JA, Peabody J (2003). Necrotizing enterocolitis among neonates in the United States. Journal of perinatology: official journal of the California Perinatal Association.

[36] Kasivajjula H, Maheshwari A (2014). Pathophysiology and current management of necrotizing enterocolitis. Indian journal of paediatrics.

[37] D’Atri F, Citi S (2002). Molecular complexity of vertebrate tight junctions (Review). Molecular membrane biology.

[38] Han X, Fink MP, Delude RL (2003). Proinflammatory Cytokines Cause No Dependent and -Independent Changes in Expression and Localization of Tight Junction Proteins in Intestinal Epithelial Cells. Shock.

[39] Hogberg N, Stenback A, Carlsson PO, Wanders A, Lilja HE (2013). Genes regulating tight junctions and cell adhesion are altered in early experimental necrotizing enterocolitis. Journal of pediatric surgery.

[40] Geuking MB, Cahenzli J, Lawson MA, Ng DC, Slack E (2011). Intestinal bacterial colonization induces mutualistic regulatory T cell responses. Immunity.

[41] Mulder IE, Schmidt B, Lewis M, Delday M, Stokes CR (2011). Restricting microbial exposure in early life negates the immune benefits associated with gut colonization in environments of high microbial diversity. PloS one.

[42] Ohnmacht C, Marques R, Presley L, Sawa S, Lochner M (2011). Intestinal microbiota, evolution of the immune system and the bad reputation of proinflammatory immunity. Cellular microbiology.

[43] Chenu JW, Cox JM (2009). Cronobacter (‘Enterobacter sakazakii’): current status and future prospects. Letters in applied microbiology.

[44] van Acker J, de Smet F, Muyldermans G, Bougatef A, Naessens A (2001). Outbreak of necrotizing enterocolitis associated with Enterobacter sakazakii in powdered milk formula. Journal of clinical microbiology.

